# Thermal and Mechanical Stress Analysis in Aircraft Hybrid Panels with Multi-Bolt Joints

**DOI:** 10.3390/ma17081872

**Published:** 2024-04-18

**Authors:** Junhua Zhang, Jie Zheng, Jianjiang Zeng, Guang Yang, Mingbo Tong

**Affiliations:** 1 College of Aerospace Engineering, Nanjing University of Aeronautics and Astronautics, Nanjing 210016, China; zjh1222@nuaa.edu.cn (J.Z.); zengjj@nuaa.edu.cn (J.Z.); 2 Department of Mechanical Engineering, University of Alberta, Edmonton, AB T6G 2R3, Canada; 3 Department of Engineering Mechanics, Tsinghua University, Beijing 100084, China; gyang4@mail.tsinghua.edu.cn

**Keywords:** composite, multi-bolt hybrid panel, thermal stress, bolt load distribution, finite element analysis

## Abstract

This study investigates the thermal stress and bolt load distribution in a hybrid panel structure of an aircraft mechanical joint under extreme temperatures. The hybrid panel structure comprises two aluminum alloy splices, six T-shaped composite stringers, and two composite skins, secured together with 96 bolts. This study analyzed the strain induced by thermal stress on composite materials and metals within the structure across temperatures, employing temperature environment tests ranging from room temperature to −54 °C, alongside a carrying capacity test at −54 °C. Furthermore, a three-dimensional simulation model of the panel structure was developed, incorporating considerations for contact, metal elastoplasticity, and the progressive damage failure of composite materials. This model facilitated the determination of thermal stress and bolt load distribution patterns. The results indicate a strong consistency between the finite element analysis outcomes and the experimental data. Temperature variations exacerbate the uneven distribution of bolt loads, concentrating the load near the edges of the hybrid structure while diminishing it in the center. The bolt load distribution parallel to the mechanical load direction forms an “M” shape, with a maximum load magnitude of approximately 31 kN. Perpendicular to the mechanical load, the bolt load undergoes significant changes, especially at the edges, reaching a maximum of about 20 kN, which warrants attention. The bolt-load distribution of the structure with the increase in mechanical load at −54 °C tends to be consistent with that at room temperature.

## 1. Introduction

Composite materials, noted for their excellent mechanical properties, including high specific strength, stiffness, fatigue resistance, and temperature tolerance, are extensively utilized in civil aircraft [1]. The amount of composite materials accounted, respectively, for 50% and 52% of the weight of the body structure in the most representative new generation of large civil aircraft (e.g., Boeing 787 and Airbus A350) [2]. The main structure of civil aircraft has evolved into composite–metal hybrid structures, including configurations like “composite panel + metal beam” and “composite panel + composite beam + metal rib” [3]. In the present study, we investigate a hybrid panel structure for next-generation large civil aircraft, consisting of two aluminum alloy splices, six T-shaped composite stringers, and two composite skins, all joined with 96 bolts.

A critical challenge in designing composite and metal hybrid structures is addressing the thermal stresses arising from the significant differences in the coefficient of thermal expansion between composite and metal materials. The coefficient of thermal expansion for metals is 10 to 20 times higher than that of composite materials. This thermal stress can sometimes constitute about 40% of the mechanical stress, leading to a stress concentration around the bolt hole area under mechanical connection conditions, thereby impacting the load-bearing capacity of the structure [4]. Hence, the design and strength analysis of composite and metal hybrid structures under thermal load have emerged as pressing issues that require immediate attention, which are investigated in the current study.

A variety of newly developed finite element models have been applied to the load distribution analysis of multi-bolt joints, considering the efficiency and the accuracy. Gray et al. [5] proposed a new bolt simulation method called global bolt joint model, which uses beam element coupling to analyze rigid surface to simulate bolts, and has the advantages of robustness, accuracy and high efficiency. Liu et al. [6] proposed an improved 2D finite element model for bolt load distribution predictions of composite multi-bolt single-lap joints, which predicts secondary bending well. Sharos et al. [7] developed a user-defined finite element capable of modeling composite joints at various loading rates, which can save a lot of computing resources. Belardi et al. [8] developed a composite joint element that can be used within a pre-existing shell model, considering the different linear and nonlinear phenomena. These models do not involve temperature changes.

Researchers have conducted studies on small-sized connectors, focusing on how ambient temperature affects connection performance [9,10,11,12,13,14,15]. Eriksson et al. [16] studied the joint structure of composite materials under thermal load by applying variational principle and complex potential theory. Kradinov et al. [17] used the same methods to study the hole side stress of bolts at any position in composite laminates under mechanical loads and uniform temperature change. They also analyzed the bolt-load distribution in single- and double-lapping joints. Ekha et al. [18,19,20] conducted several studies on secondary bending and bolt-load distribution through tests and finite element analysis. The results showed that bolt diameter, bolt hole clearance and temperature greatly influenced load distribution. Yang et al. [21] derived the bolt-load calculation formula of hybrid structure connectors under the action of temperature, carried out the temperature field test of composite–metal panel structure in different structural forms and obtained the U-shaped distribution rule of bolt-load. Lei et al. [22,23] analyzed the influence of parameters such as the quantity and spacing of bolts on the bolt-load distribution in different structural forms for a single-lap multi-bolt hybrid structure, and revealed the rule that the maximum bolt-load is limited by the structural size effect. Kapidzic et al. [24] studied the bolt-load distribution of wing box structure under bending load and temperature change by using 2D and 3D models. At present, there are few investigations on thermal stress of hybrid structures from the published research data. The experimental studies mainly focus on the small-size connection structure with single row of bolts and the research on the complex large-size structure of multi-row and multi-row of bolts is not involved. The simulation analysis mainly considers the elastic behavior of the material and the simplified bolt model, regardless of the influence of metal plasticity and composite damage. In addition, the magnitude of thermal stress of hybrid structure is affected by the size effect and the maximum bolt load tends to stabilize after a rapid increase with the increase in the length and the number of screws.

It is more representative to carry out research on thermal stress and bolt-load distribution of large-size hybrid structures of the new generation civil aircraft. In this study, the specimen of the multi-bolt hybrid panel structure is described in Section 2, and the finite element model is established, considering contact, metal elastoplasticity, and the progressive damage failure of composite materials, in Section 3. The design of temperature environment test and the test result compared with that by simulation are drawn in Section 4 and Section 5. The influence of temperature change and mechanical load change on bolt load distribution is discussed in Section 6. Finally, several conclusions are drawn in Section 7.

## 2. The Hybrid Panel Structure

**Figure 1:**
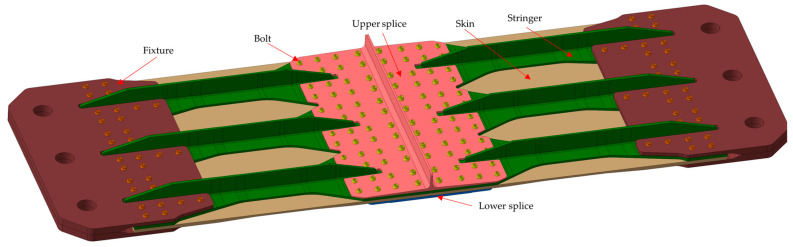
The butt joint specimen of hybrid panel structure in the center wing.

**Figure 2:**
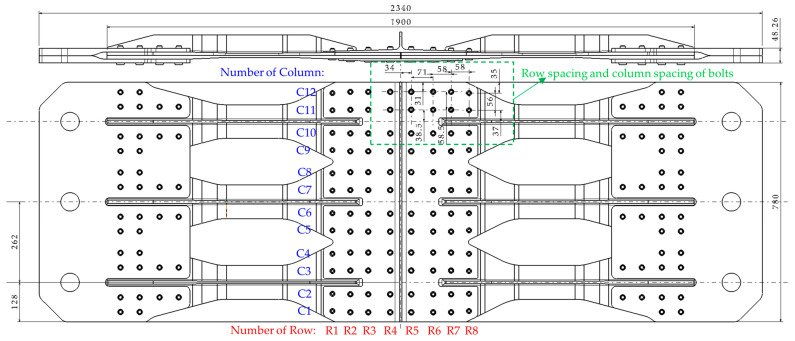
General assembly diagram of hybrid panel structure (unit: mm).

**Figure 3:**
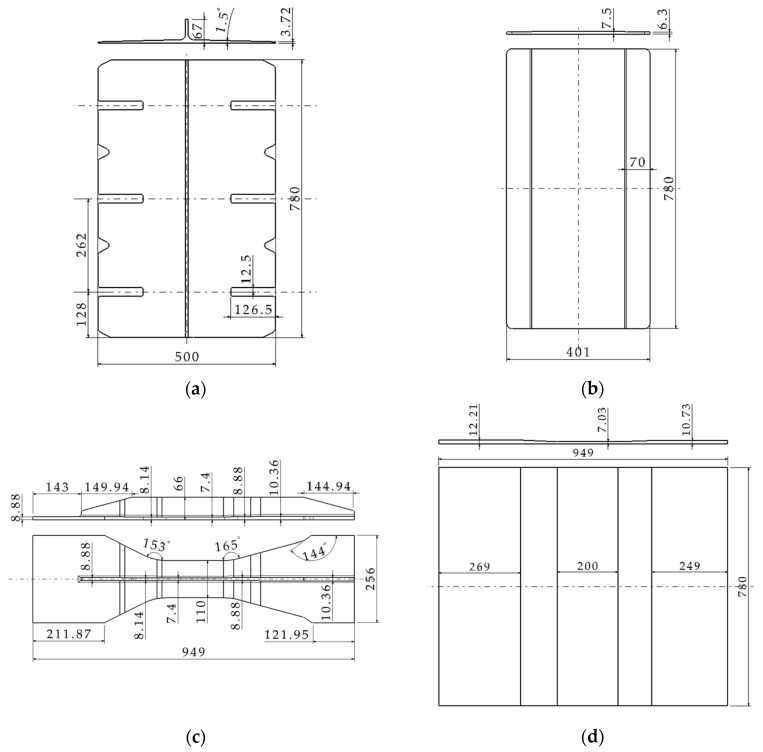
The shapes and dimensions of (**a**) the upper splice, (**b**) the lower splice, (**c**) the stringer, and (**d**) the skin (unit: mm).

This study focuses on a multi-bolt hybrid panel structure in an aircraft center wing, which is a scheme in the design stage of a certain type of aircraft, as shown in Figure 1 and Figure 2. The hybrid panel consists of two aluminum alloy splices (upper and lower), six T-shaped composite stringers, and two composite skins, interconnected by 96 bolts arranged in 8 rows and 12 columns. The bolt number and specific row and column spacing are shown in Figure 2. The bolts in R1 and R8 are single shear with a diameter of 11.1125 mm. The bolts in the remaining six rows feature double shear, with diameters of 12.7 mm in rows R2, R3, R6, and R7, and 14.2875 mm in rows R4 and R5. Figure 3 shows the shapes and dimensions of all parts. Both skin and stringer are made of composite prepreg T800 with a single layer thickness of 0.185 mm. According to the quasi-isotropic principle, the specific ply information is listed in Table 1.

**Figure 4:**
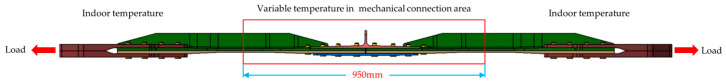
Mechanical and temperature loads of hybrid panel structure.

Targeting the thermal stress analysis of the hybrid structure, such as the wall panel, a test is designed under a 600 kN mechanical load and at a low temperature of −54 °C, covering a 950 mm range within the mechanical connection area, as illustrated in Figure 4. The material properties provided by Commercial Aircraft Corporation of China are shown in Table 2 and Table 3. In this study, the temperature induced modulus and thermal expansion coefficient are not considered in the analysis because they do not change significantly at −54 °C [25]. The specific parameters are shown in Table A1 of Appendix A.

## 3. Finite Element Simulation of Hybrid Panel under Temperature Field

**Figure 5:**
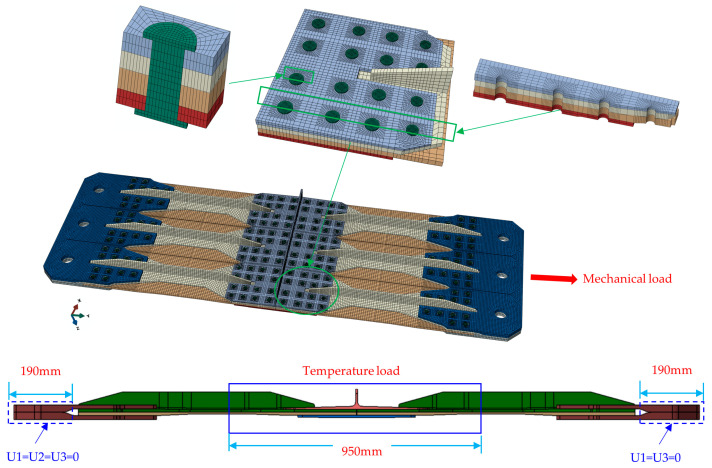
Finite element model of the hybrid panel structure.

Due to the thermal expansion coefficient of various materials in the panel structure and the size effect of the structure, this study establishes a three-dimensional finite element solid model based on ABAQUS to simulate the stress caused by temperature load, as shown in Figure 5. SC8R continuous shell elements were used to discretize the composite stringer and skin, C3D8I three-dimensional stress elements were used to simulate the upper and lower splices and bolts. The mesh size of the hole edge was selected at least 1.2 mm after the mesh independence verification, considering computational accuracy and resources, and the model calculation results are listed in Table A2 of Appendix A.

**Figure A1:**
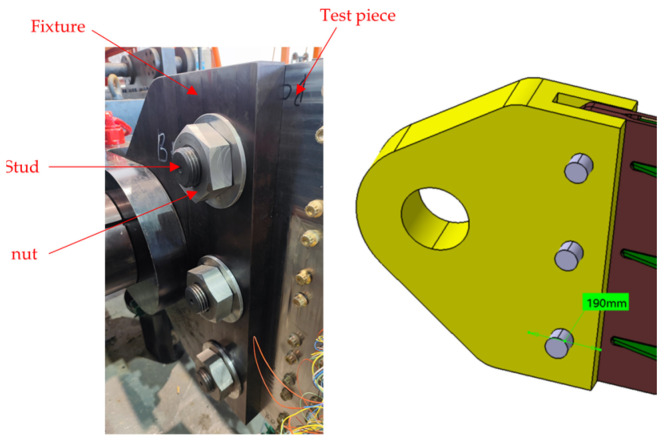
Photograph of the installation condition in the end of the hybrid panel.

The factors such as contact, metal elastoplasticity and progressive damage failure of composite materials were considered in the model, and the details are shown in Appendix B. According to the literature [19], the fastener preload and friction coefficient have little influence on the connection performance. The preload values to the bolts of different diameters are listed in Table A3 of Appendix A. Regardless of the influence of the fastener preload in the model, the friction coefficient is set at 0.1 [26,27], which is referred to the composite–metal contact, and the contact property adopts the hard contact and Coulomb friction model. The failure mode of composite monolayers is simulated by the Hashin criterion [28,29,30], and the constitutive relation of metal materials is obtained by material test. Simple support constraints (U1 = U2 = U3 = 0) were applied to the upper and lower surfaces of one end of the steel fixture, and displacement constraints (U1 = U3 = 0) were applied to the upper and lower surfaces of the other end. The installation condition in both ends of hybrid panel are shown in Figure A1 of Appendix A. Mechanical load was simulated using a concentrated load, while the temperature field was modeled through a predefined field in ABAQUS.

## 4. Experimental Design of Hybrid Panel

### 4.1. Test Design

Figure 1 shows the size of the test part, and the materials of each component are listed in Table 1, Table 2 and Table 3. The testing setup comprises a temperature-controlled cabinet, static testing machine, strain gauges, strain measurement and acquisition equipment, thermocouples, supporting inspection instruments, heat compensation plates, and auxiliary fixtures.

**Figure 6:**
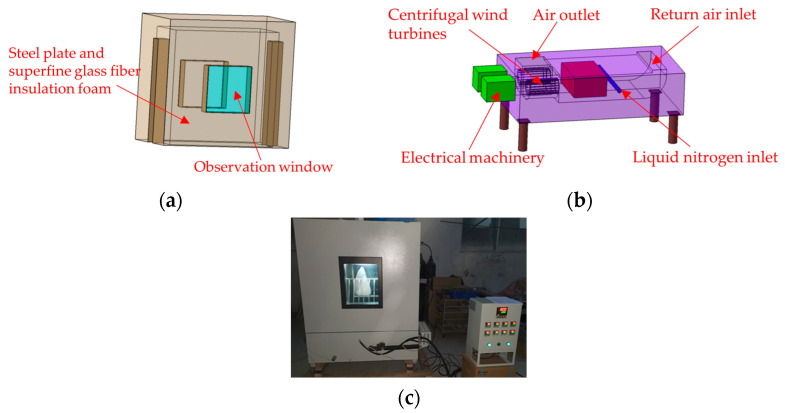
(**a**) Upper part of temperature-controlled chamber; (**b**) lower part of temperature-controlled chamber; (**c**) photograph of temperature-controlled chamber.

A temperature-controlled chamber, with its main structure depicted in Figure 6, was designed to accommodate the test requirements for a temperature drop to −54 °C within the test area. The inside and outside box of the temperature chamber are made of 304SUS high-grade stainless steel. The thermal insulation material between the inner and the outer boxes is high-quality ultra-fine glass fiber thermal insulation foam, and a silicone sealing structure is used between the door and the door frame. Temperature reduction is achieved through the vaporization of liquid nitrogen, with double fans facilitating circulation to ensure uniform temperature distribution throughout the chamber. The temperature box and studio sizes are 1150 mm × 600 mm × 1380 mm and 950 mm × 400 mm × 980 mm (length × width × height), respectively. The temperature fluctuation is less than ±2 °C, the temperature uniformity is less than ±2 °C. 

**Figure 7:**
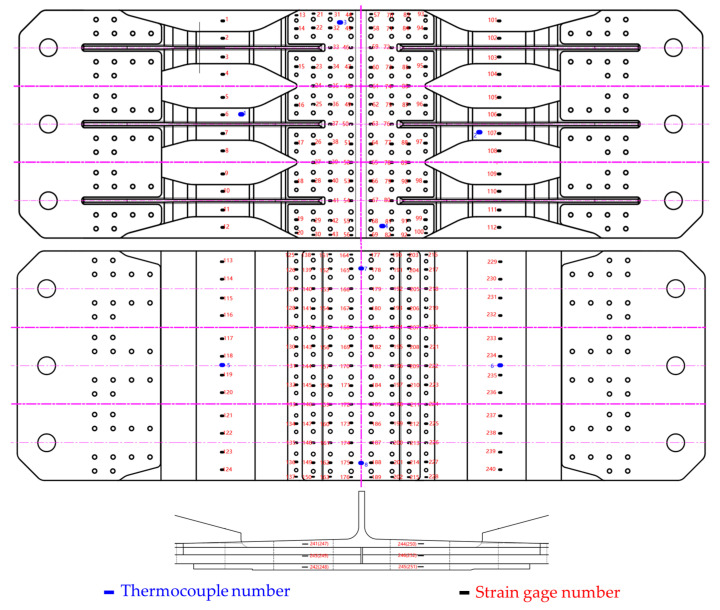
Distribution of thermocouple and strain gage in hybrid panel.

Temperature-resistant strain gauges are affixed to composite and metal plates, employing a temperature compensation method to nullify the strain indications resulting from the strain gauge’s temperature effects. Eight thermocouples are strategically placed on both composite and metal plates to monitor the surface temperature of the test specimens, taking into account the non-uniformity of convection and the necessity for temperature compensation. The location and number of strain gauge and temperature are shown in Figure 7.

**Figure 8:**
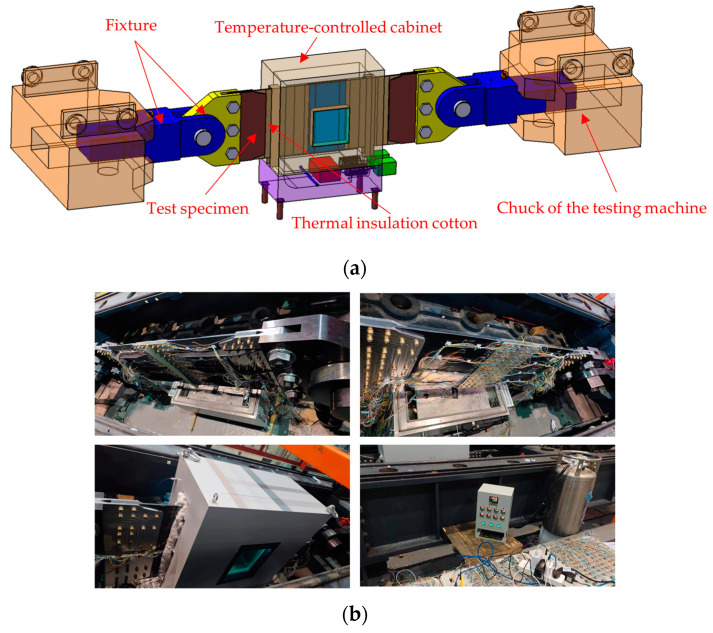
(**a**) Schematic diagram of the test setup; (**b**) photograph of the test setup.

The test was carried out on the 1000 t horizontal test machine for the space and load requirements of the test, as shown in Figure 8. The test piece is connected with the chuck of the testing machine through an auxiliary fixture. In the left and right gaps between the specimen and the temperature box, thermal insulation cotton is filled to ensure that the temperature of the test area meets the test requirements.

### 4.2. Test Procedure

After installing the test specimen and completing the preparatory work, a 600 kN mechanical load is applied to eliminate the initial assembly gap between the test piece and the fixture. Subsequently, with the mechanical load held constant, low temperature tests are conducted at various temperatures, including 10 °C (room temperature), −10 °C, −30 °C, and −54 °C. Upon reaching the working temperature, the system was maintained at this temperature level for approximately two hours, during which strain data was collected at 30 s intervals. The bearing capacity test of structure was carried out at −54 °C in the last set of tests.

## 5. Comparison between Measured Results and Simulation Results

**Figure 9:**
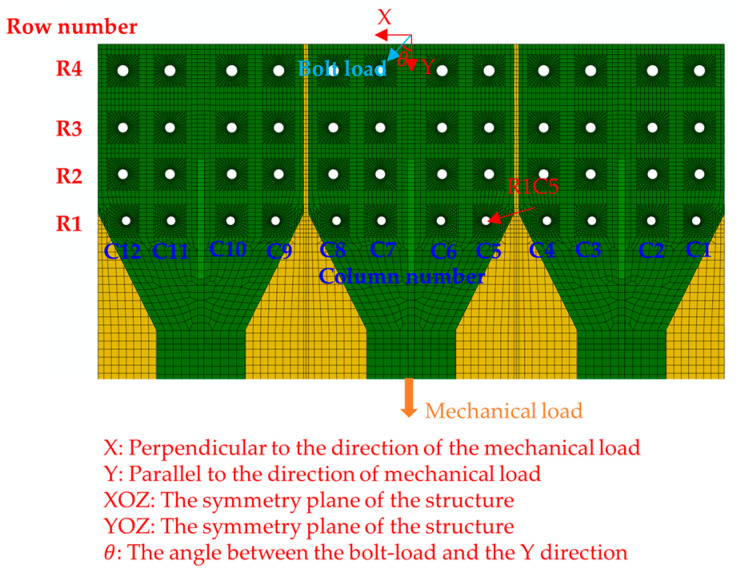
Diagram of bolt number and bolt-load direction.

In this paper, the bolts are numbered to study their load distribution. For example, R1C5 indicates the bolt in row 1 and column 5, R1 indicates the first row, and C2 indicates the second column, as shown in Figure 9. The bolt load in the wall panel structure mainly bears the shear load, and it is divided into X and Y two directions. The X direction indicates that the direction of the bolt load component is perpendicular to the direction of the mechanical load and toward the interior of the structure. The Y direction indicates that the direction of the bolt load component is parallel to the direction of the mechanical load and along the direction of loading.

### 5.1. Strain Distribution with the Decrease in Temperature

The strain data of the panel test specimen fluctuates within 10 μ at each temperature point, and the data from the thermocouple show fluctuations within 2 °C at each position, indicating the test specimen’s temperature has stabilized. “μ” is a dimensionless unit of strain measurement.

Taking into account the variability in the testing process and the inherent symmetry of the structure, the collected strain data are appropriately processed. The strain data is set as 0 μ under 600 kN mechanical load at 10 °C. Then, after the 3σ criterion to determine whether the data is valid, a group of symmetrical strain gauge measurements in one test are averaged, and the strain data of the three groups of low temperature tests are averaged. For example:

(1) $$\overset{-}{\varepsilon_{T}\left(1\right)}=\frac{1}{3}\sum_{i=1}^{3}(\frac{1}{4}(\varepsilon_{T,i}\left(1\right)+\varepsilon_{T,i}\left(12\right)+\varepsilon_{T,i}\left(101\right)+\varepsilon_{T,i}\left(112\right))),$$

where $\overset{-}{\varepsilon_{T}\left(1\right)}$ is the treated value of strain gage 1 in the quarter of the structure at T °C, $\varepsilon_{T,i}\left(1\right)$ is the value measured by strain gage 1 at the ith temperature test at T °C, $\varepsilon_{T,i}\left(12\right)$, $\varepsilon_{T,i}\left(101\right)$ and $\varepsilon_{T,i}\left(112\right)$ are similar to $\varepsilon_{T,i}\left(1\right)$.

**Figure 10:**
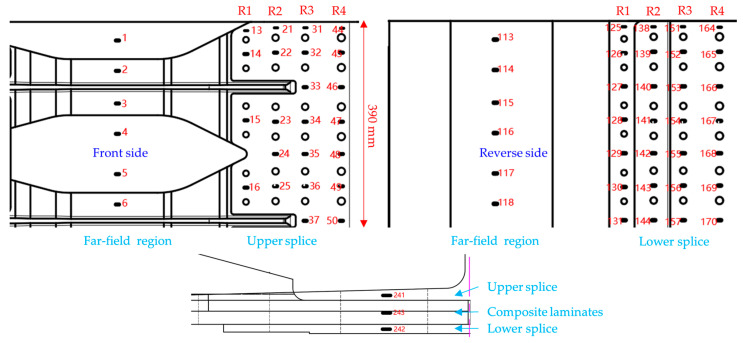
Strain gages in the quarter of the structure.

Because of symmetry, only one quarter of the structure is discussed in this article, as shown in Figure 10.

**Figure 11:**
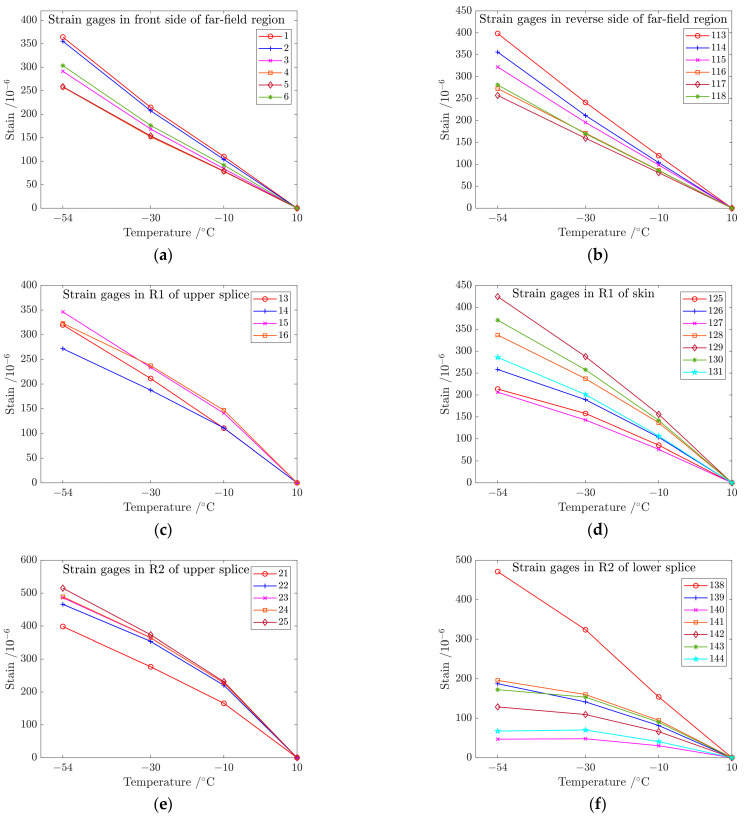

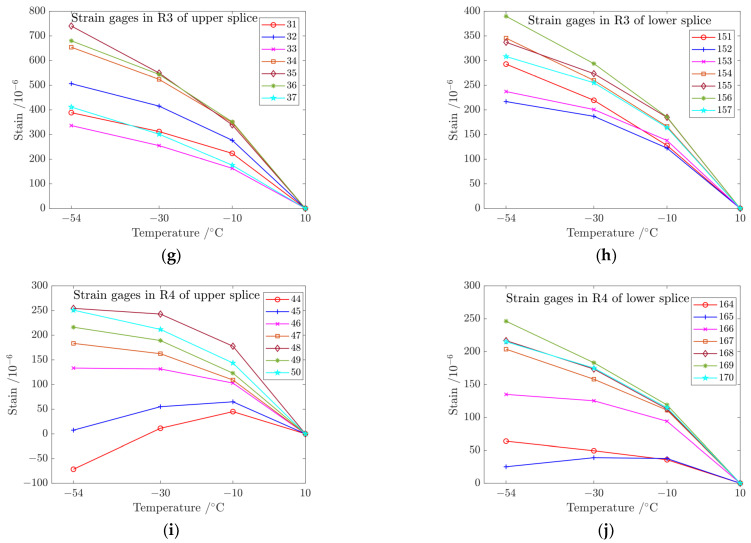
Test values of strain gages in (**a**) front side of far-field region, (**b**) reverse side of far-field region, (**c**) R1 of upper splice, (**d**) R1 of lower splice, (**e**) R2 of upper splice, (**f**) R2 of lower splice, (**g**) R3 of upper splice, (**h**) R3 of lower splice, (**i**) R4 of upper splice, and (**j**) R4 of lower splice.

Figure 11 presents the measured strains generated by thermal stress at each test point with four different temperatures of 10 °C, −10 °C, −30 °C, and −54 °C. The values of strain gages demonstrate a linear increase with the decrease in temperature in the front and reverse sides of the far-field region. 

The changes in strain values are most significant in R3 and least in R4 among the upper and lower metal splices, exhibiting good linearity in R1, R2, and R3, with less linearity observed in R4. The strain values maintain a linear relationship with temperature changes, attributed to the constant coefficient of thermal expansion of the material within this temperature range and the structure’s stress level remaining within the elastic range under the applied load.

**Figure 12:**
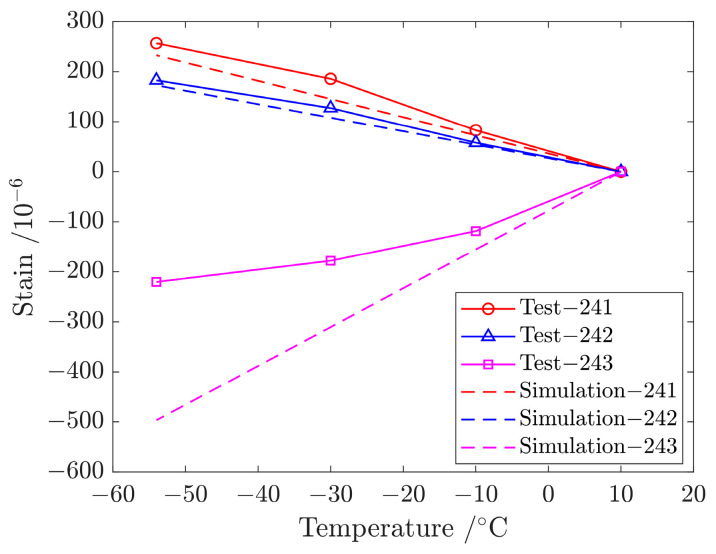
Test value and simulation result of strain gages (241, 242 and 243) in upper splice, lower splice and composite laminates at different temperatures.

The thermal expansion coefficient of the metal plate is greater than that of the composite plate, resulting in tension of the metal splices (lower and upper) and compression of the composite plate in the connection area with the decrease in temperature, as shown in Figure 12. The simulated values are in good agreement with the test values on the metal plate, but the difference is large on the composite laminates because there is a layer of thermoplastic on the surface of the co-curing composite girder and skin.

### 5.2. Strain Distribution Included by Thermal Stress at a Working Temperature of −54 ℃

**Figure 13:**
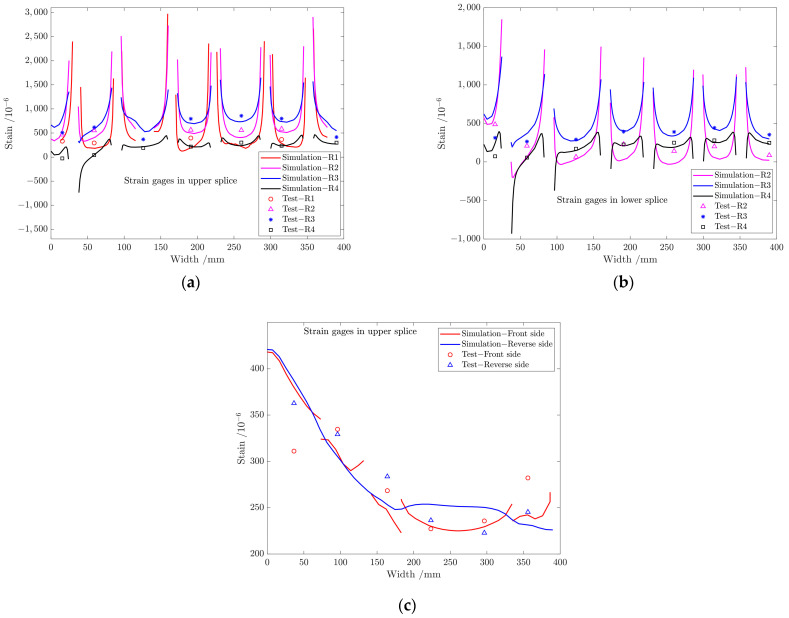
Comparison between simulation and test values of strain gages in (**a**) front side of upper splice, (**b**) reverse side of lower splice, and (**c**) front and reverse side of far-field region. All the simulation and experimental temperatures are at a working temperature of −54 °C.

The finite element simulation outcomes and experimental data from all measurement points are compared at −54 °C, as shown in Figure 13. The distribution of strain values in Figure 13a,b shows consistency between simulation and test results in the metal splices, with a gradual decrease observed through the rows R3, R2, R1, and R4 in the upper splice and through the rows R3, R1 and R2 in the lower splice, which presents an “*M*”-shaped distribution from R1 to R8 of the whole structure. Figure 13c shows that the strain difference between simulation and test results in the far field region of the structure is slightly larger, which is caused by these strain gauges being close to the gradient region of the room temperature environment to −54 °C temperature environment. Overall, the simulation results agree with the test values.

### 5.3. Fracture Load and Mode at a Working Temperature of −54 ℃

**Figure 14:**
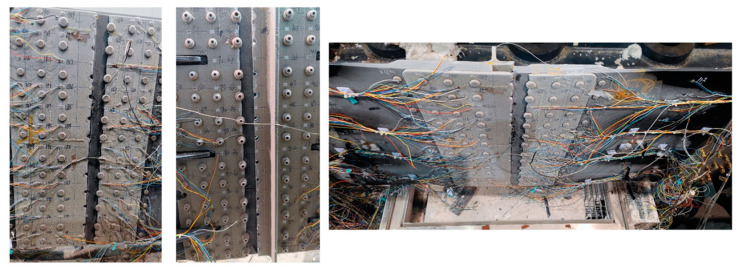
Damage morphology of the test piece at −54 °C.

**Figure 15:**
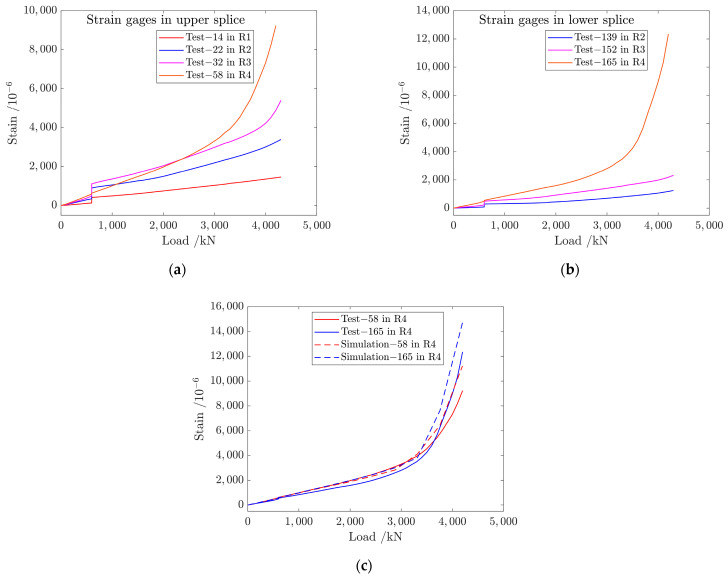
Strain–load curves of strain gages in (**a**) upper splice and (**b**) lower splice at −54 °C; (**c**) comparison between simulation and test strain–load curves of strain gages 58 and 165.

**Figure 16:**
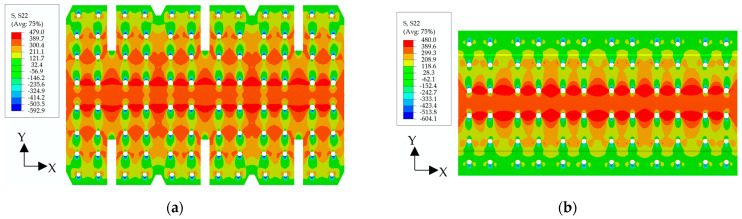
Computed stress distribution of (**a**) upper splice and (**b**) lower splice, when the mechanical load is 4214 kN at −54 °C.

Figure 14 shows that the experimental failure load of the structure is 4402 kN at −54 °C and the test fracture mode is that the mental splice is stretched and broken in R4 or R5. The strain–load curves of strain gages in the lower and upper splices are shown in Figure 15a,b. When the mechanical load is greater than 3000 kN, the values of strain gages in R3 and R4 of two metal splices begin to appear nonlinear. With the increase in external load, the values of strain gages in R4 are the largest. Meanwhile, the hole edge stress in R4 reaches the ultimate strength of the material under the combined action of nailing load and bypass load and metal splice is broken by pulling. Test results agree with the simulation results that the fracture load is 4214 kN, as shown in Figure 15c and Figure 16.

## 6. Effects of Temperature and Mechanical Load on Bolt Load Distribution of Hybrid Panel

### 6.1. Analysis of Bolt-Load Distribution at a Working Temperature of −54 ℃

Utilizing the finite element model described in Section 3, an external load of 600 kN is maintained constant, and the room temperature is set at 10 °C. Subsequently, the temperature in the mechanical connection area is reduced to −54 °C.

Table 4 and Table 5 show the bolt load distribution of hybrid panel structure at 10 °C and −54 °C. The load direction of all bolts is almost the same as the loading direction, and the load value in each row is close and uniform at 10 °C. The bolt-load distribution at −54 °C is symmetrical in the X and Y direction of the hybrid panel structure. It indicates that the internal force caused by the thermal stress is balanced inside the connection area in the cooling stage due to the different coefficients of thermal expansion between the metal plate and the composite plate. The total temperature load is zero externally in the connection area. The load distribution of bolts in each row is not uniform.

**Figure 17:**
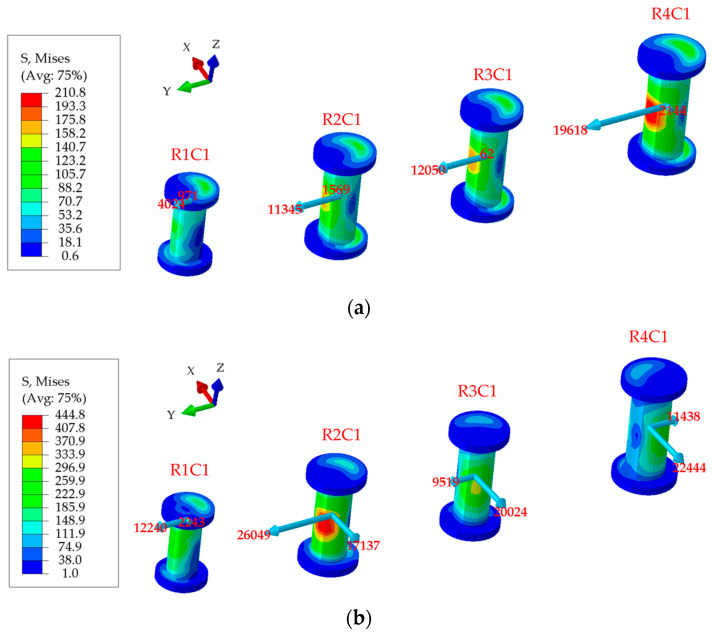
(**a**) Load on bolts in C1 at 10 °C; (**b**) load on bolts in C1 at −54 °C.

Figure 17 shows that the load direction of bolts in C1 of the panel structure is at a certain angle to the loading direction, indicating that the bolt load caused by temperature change has an X-direction component at −54 °C. In Table 5, the bolt-loads of four columns, including C1, C2, C11 and C12, are significantly greater than that of other columns, and the angles in these four columns are more than 30 °C, which indicates that close to the edge of the structure, there is a higher load.

**Figure 18:**
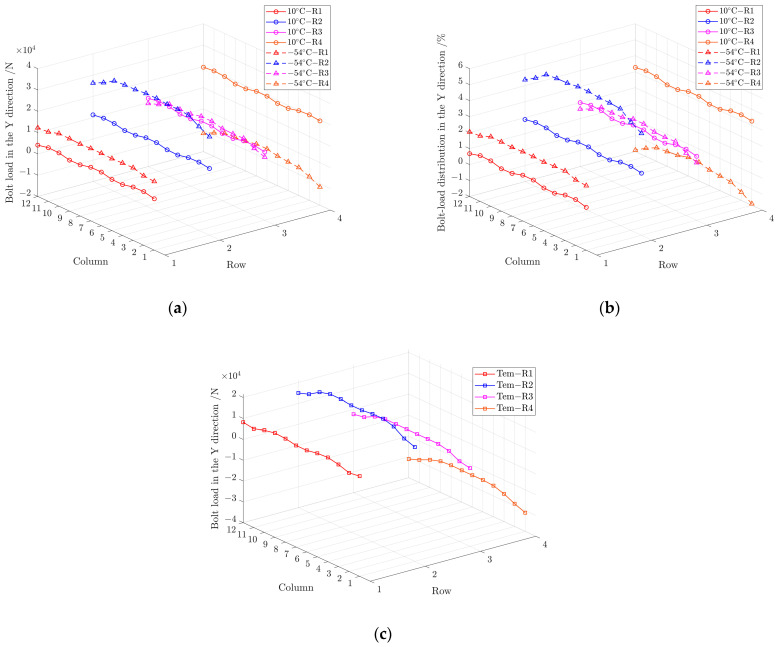
(**a**) Load on bolts in the Y direction at 10 °C and −54 °C; (**b**) load distribution of bolts in the Y direction at 10 °C and −54 °C; (**c**) load on bolts with temperature included load in the Y direction from 10 °C to −54 °C.

Figure 18 shows the corresponding bolt-load ratio in the Y direction at 10 °C and −54 °C when the external load is 600 kN. There is a large difference in the bolt-load ratio at 10 °C and −54 °C. At 10 °C, the bolt-load distribution ratios across rows R1, R2, R3, and R4 are uniform, approximately 0.8%, 1.9%, 1.9%, and 3.1%, respectively. The bolt-load distribution of the entire structure exhibits a “∩”-shaped distribution from R1 to R8. However, the bolt-load proportion increases to 2.3% in R1, significantly increases to 5.4% in R2, reduces slightly to 1.6% in R3, and decreases greatly to −1.5% in R4 as the temperature drops to −54 °C, which presents a “M”-shaped distribution from R1 to R8 of the whole structure. In addition, the maximum bolt-load magnitude is about −31 kN when the thermal load is included and appears on R4C1 and R4C12. It indicates that the temperature change will aggravate the uneven distribution of the bolting load in the Y direction of the structure, which has a greater impact on the edge of the structure and a smaller impact on the middle part.

**Figure 19:**
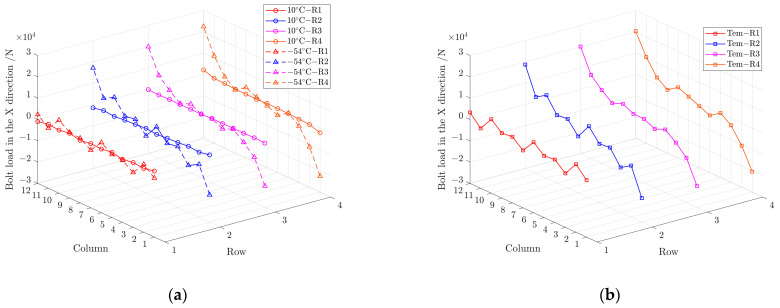
(**a**) Load on bolts in the X direction at 10 °C and −54 °C; (**b**) load on bolts with temperature included load in the X direction from 10 °C to −54 °C.

Figure 19 shows the corresponding load on bolts in the X direction at 10 °C and −54 °C when the external load is 600 kN. The value of bolt load is small and below 1 kN on all bolts, except for the bolts in C1 and C12 near the end of the structure, which is slightly higher to about 2 kN at 10 °C. When the temperature drops to −54 °C, the bolt load caused by temperature variation changes little in the middle part of the structure. The load of the double shear bolts in C1, C2, C11 and C12 changes significantly, and the maximum bolt-load magnitude caused by temperature change reaches −20 kN, which cannot be ignored. The result indicates that the temperature included bolt load in X direction when the thermal load is included is larger close to the edge of the structure is, and has less effect in the middle area.

### 6.2. Influence of Temperature Change on Bolt-Load Distribution

**Figure 20:**
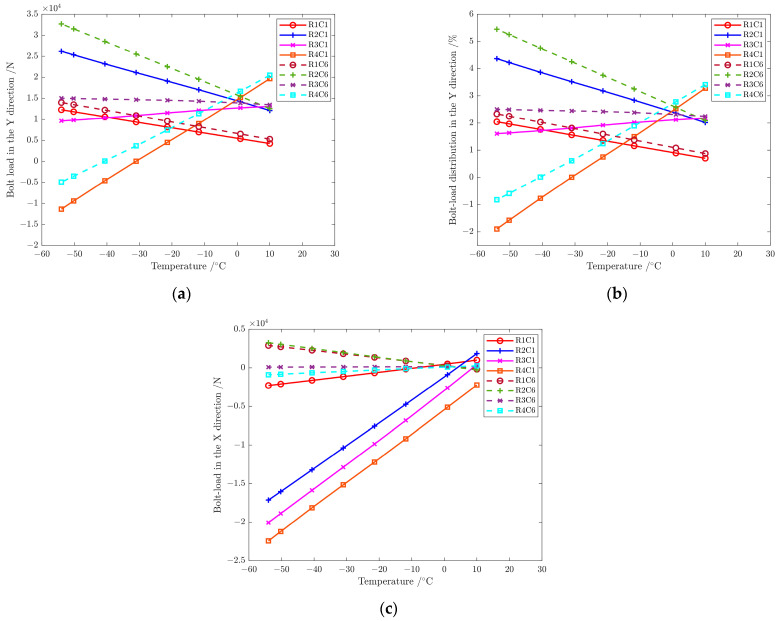
(**a**) Load on bolts in the Y direction at different temperatures; (**b**) load distribution of bolts in the Y direction at different temperatures; (**c**) load on bolts in the X direction at different temperatures.

The influence of the temperature change was investigated with the finite element model in Section 3, keeping the 600 kN mechanical load unchanged and studying eight working temperatures of 10 °C, 0 °C, −10 °C, −20 °C, −30 °C, −40 °C, −50 °C and −54 °C. According to the analysis in Section 6.1, the load on bolts near the end and middle part of the structure varies greatly when the temperature decreases. Then, bolts in C1 and C6 are selected for discussion in this section. Figure 20 shows the load distribution of bolts in C1 and C6 at different temperatures when the external load is 600 kN.

The bolt load in each row changes differently in the Y direction with the decrease in temperature, gradually increasing in R1 and R2, changing slightly in R3, and gradually decreasing in R4. It indicates that the mechanical load is strengthened by the temperature load in R1 and R2 and weakened in R4 when the temperature drops, while the temperature load has little influence on the mechanical load in R3. In addition, the bolt load of R4C1 and R4C6 is reduced to 0 kN at about −30 °C and −40 °C, respectively, and the load direction begins to be opposite to the mechanical load direction. The temperature change has little effect on the load on bolts in C6 and the single shear bolt R1C1 in the X direction. The load of three double-shear bolts in C1 gradually decreases as the temperature drops, and the amplitude of load variation with temperature is close and −320 N/°C.

### 6.3. Influence of Mechanical Load Change on Bolt-Load Distribution

Using the finite element model in Section 3, the mechanical load was first applied to 600 kN, the ambient temperature remained unchanged at −54 °C, and the external load was continued. In this section, bolts in R1 and R6 are chosen for discussion; the nonlinear behavior of the structure is measured by the ratio of the applied mechanical load $F_{g}$ to the failure load $F_{b}$ of the structure, with $F_{b}=4402\;kN$.

**Figure 21:**
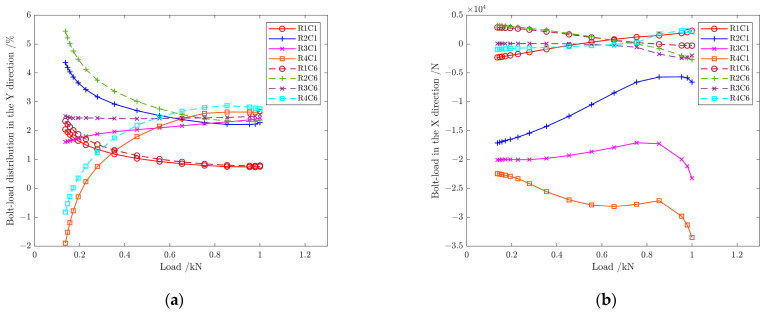
(**a**) Load distribution of bolts in the Y direction as mechanical load increasing at −54 °C; (**b**) load on bolts in the X direction as mechanical load increasing at −54 °C.

Figure 21 shows the bolt-load distribution in C1 and C6 in the process of mechanical load application at −54 °C. When the external load is 600 kN ($F_{g}/F_{b}=13.2\%$), the bearing capacity of each bolt varies greatly. The load proportion differs from each bolt with the increase in mechanical load in the Y direction, which increases gradually on R4C1 and R4C6 bolts, increases slightly on R3C1 and R3C6 bolts, and decreases gradually on R1C1, R1C6, R2C1 and R2C6 bolts, as shown in Figure 21a. When the ratio $F_{g}/F_{b}=21.1\%$, the load direction of all bolts is the same. The plastic strain near the bolt hole accumulates, and the proportion of each bolt load tends to stabilize as the load continues to increase. When the ratio $F_{g}/F_{b}\geq 75\%$, the load ratio is 2.7% in R4, close to 0.8% in R1, 2.3% in R2, and 2.2% in R3, respectively. When the ratio $F_{g}/F_{b}\geq 85\%$, the bolt-load distribution ratio on each row is evenly distributed again, which is similar to the “∩”-shaped distribution at 10 °C. Figure 21b shows that the bolt load in the X direction changes little in C6 at the middle part of the structure and on the single shear bolt R1C1. However, the load of the double-shear bolts changes greatly in C1 near the edge of the structure. With the increase in external load, the load on the bolt increases gradually on R2C1, is almost unchanged on R3C1, and decreases gradually on R4C1. The load of three bolts is still negative, which indicates the load direction is toward the outside of the structure. When the ratio $F_{g}/F_{b}\geq 85\%$, a load drop phenomenon occurs in three bolts.

## 7. Conclusions

1. In this study, we conducted thermal and mechanical tests on a multi-bolt hybrid panel structure. The strain values of each test point at different temperatures indicates that the strain values and temperature change maintain a linear relationship as expected. The failure load of the structure is 4402 kN.

2. A three-dimensional finite element model is established considering contact and nonlinearity of materials. The simulation results agree with the test values and according with the experimental rules.

3. In the multi-bolt hybrid panel structure for next-generation civil aircraft, thermal stress is self-balanced within the connection area, yet temperature variations exacerbate the uneven distribution of bolt load. The load is focused on the bolts near the structure’s edge and diminishes in the center.

4. In this study, the bolt-load distribution parallel to the mechanical load direction adopts an “M” shape at −54 °C, contrasting with the “∩” shape observed at room temperature. Perpendicular to the mechanical load direction, the bolt load undergoes its most significant changes at the structure’s edge. The maximum load magnitudes in two directions, when the thermal load is included, are about 31 kN and about 20 kN, respectively. The bolt-load distribution of the structure tends to be consistent with that at room temperature with the increase in mechanical load at −54 °C. In the structural design, the size of bolts near the end of the structure should consider the additional load caused by temperature changes to prevent catastrophic damage at the end.

## Figures and Tables

**Table 1:** Information of composite laminates.

Part	Thickness of Region/mm	Ply Information
Skin	12.21	[45/90/−45/90/−45/0/45/0/−45/0/45/0/0/−45/0/0/45/0/0/45/0/0/−45/0/0/45/0/0/−45/−45/0/0/45]_s_
	7.03	[45/90/−45/90/−45/0/0/45/0/0/45/0/0/−45/0/0/−45/0/45]_s_
	10.73	[45/90/−45/90/−45/0/45/0/−45/0/45/0/0/−45/0/0/45/0/0/45/0/0/−45/0/0/−45/0/0/45]_s_
Stringer	10.36	[45/90/−45/0/0/−45/0/0/45/90/45/0/0/−45/0/0/−45/0/0/45/0/45/0/−45/0/−45/0/45]_s_
	8.88	[45/90/−45/0/0/−45/0/0/45/90/45/0/0/−45/0/0/−45/0/45/0/0/−45/0/45]_s_
	7.4	[45/90/−45/0/0/−45/0/0/45/0/0/−45/0/0/45/0/0/−45/0/45]_s_
	8.14	[45/90/−45/0/0/−45/0/0/45/90/45/0/0/−45/0/0/45/0/0/−45/0/45]_s_

**Table 2:** Material properties of T800.

$E_{1}$/MPa	$E_{2}$/MPa	$\mu_{12}$	$G_{12}$/MPa	$\alpha_{1}$/($10^{-6}\cdot {{}^{\circ}C}^{-1}$)	$\alpha_{2}$/($10^{-6}\cdot {{}^{\circ}C}^{-1}$)	$\alpha_{3}$/($10^{-6}\cdot {{}^{\circ}C}^{-1}$)
163,500	9000	0.319	4140	3.5	32	32

**Table 3:** Material properties of 2024-T351, Ti-6Al-4V and 4130.

Part	Material	E/MPa	$\sigma_{\mathrm{ys}}$/MPa	$\sigma_{b}$/MPa	ψ/%	μ	α$/\;(10^{-6}\cdot {{}^{\circ}C}^{-1}$)
Splice	2024-T351	73,777	344.7	475.2	12	0.33	22
Bolt	Ti-6Al-4V	116,521	820.5	951.5	10	0.31	8.8
Fixture	4130	199,810	985.9	1031.8	12	0.32	11

**Table 4:** The value and the angle of bolt load in hybrid panel structure at 10 °C.

Row Number		Column Number											
		C1	C2	C3	C4	C5	C6	C7	C8	C9	C10	C11	C12
R1	load/kN	4.1	5.1	5.0	3.9	4.0	5.1	5.1	4.0	3.9	5.0	5.1	4.1
	θ/°	13.5	−0.5	4.9	−5.3	9.1	−1.9	1.9	−9.1	5.3	−4.9	0.5	−13.5
R2	load/kN	11.4	12.1	11.9	10.8	11.0	12.1	12.1	11.0	10.8	11.9	12.1	11.4
	θ/°	7.9	2.8	4.7	2.5	1.0	−1.2	1.2	−1.0	−2.5	−4.7	−2.8	−7.9
R3	load/kN	12.1	12.9	12.7	11.5	11.6	12.8	12.8	11.6	11.5	12.7	12.9	12.1
	θ/°	0.3	1.2	0.5	1.0	0.3	0.6	−0.6	−0.3	−1.0	−0.5	−1.2	−0.3
R4	load/kN	19.7	20.2	19.8	18.6	18.6	19.8	19.8	18.6	18.6	19.8	20.2	19.7
	θ/°	−6.2	−1.6	−1.0	0.1	0.1	0.7	−0.7	−0.1	−0.1	1.0	1.6	6.2
R5	load/kN	19.7	20.2	19.8	18.6	18.6	19.8	19.8	18.6	18.6	19.8	20.2	19.7
	θ/°	−173.8	−178.4	−179.0	179.9	179.9	179.3	−179.3	−179.9	−179.9	179.0	178.4	173.8
R6	load/kN	12.1	12.9	12.7	11.5	11.6	12.8	12.8	11.6	11.5	12.7	12.9	12.1
	θ/°	179.7	178.8	179.5	179.0	179.7	179.4	−179.4	−179.7	−179.0	−179.5	−178.8	−179.7
R7	load/kN	11.4	12.1	11.9	10.8	11.0	12.1	12.1	11.0	10.8	11.9	12.1	11.4
	θ/°	172.1	177.2	175.3	177.5	179.0	−178.8	178.8	−179.0	−177.5	−175.3	−177.2	−172.1
R8	load/kN	4.1	5.1	5.0	3.9	4.0	5.1	5.1	4.0	3.9	5.0	5.1	4.1
	θ/°	166.5	−179.5	175.1	−174.7	170.9	−178.1	178.1	−170.9	174.7	−175.1	179.5	−166.5

**Table 5:** The value and the angle of bolt load in hybrid panel structure at −54 °C.

Row Number		Column Number											
		C1	C2	C3	C4	C5	C6	C7	C8	C9	C10	C11	C12
R1	load/kN	12.4	12.7	14.7	14.0	13.8	14.2	14.2	13.8	14.0	14.6	12.7	12.4
	θ/°	−10.4	8.1	−16.6	−3.7	−1.4	11.6	−11.6	1.5	3.7	16.6	−8.1	10.4
R2	load/kN	31.2	29.0	32.6	32.1	32.2	32.8	32.8	32.2	32.1	32.6	29.0	31.2
	θ/°	−33.3	−10.4	−14.1	−2.6	−3.9	5.7	−5.7	3.8	2.6	14.0	10.4	33.3
R3	load/kN	22.2	14.6	14.5	13.7	14.1	15.0	15.0	14.1	13.7	14.5	14.6	22.2
	θ/°	−64.6	−37.9	−17.0	−1.3	−9.9	0.3	−0.3	9.9	1.3	17.0	37.9	64.6
R4	load/kN	25.2	14.1	7.6	6.4	6.7	5.0	5.0	6.7	6.4	7.6	14.1	25.2
	θ/°	−117.0	−129.0	−152.7	177.5	−152.7	−169.7	170.0	152.9	−177.2	152.9	129.1	117.0
R5	load/kN	25.2	14.1	7.6	6.4	6.7	5.0	5.0	6.7	6.4	7.6	14.1	25.2
	θ/°	−63.0	−51.0	−27.3	2.5	−27.3	−10.3	10.0	27.1	−2.8	27.1	50.9	63.0
R6	load/kN	22.2	14.6	14.5	13.7	14.1	15.0	15.0	14.1	13.7	14.5	14.6	22.2
	θ/°	−115.4	−142.1	−163.0	−178.7	−170.1	179.7	−179.7	170.1	178.7	163.0	142.1	115.4
R7	load/kN	31.2	29.0	32.6	32.1	32.2	32.8	32.8	32.2	32.1	32.6	29.0	31.2
	θ/°	−146.7	−169.6	−165.9	−177.4	−176.1	174.3	−174.3	176.2	177.4	166.0	169.6	146.7
R8	load/kN	12.4	12.7	14.7	14.0	13.8	14.2	14.2	13.8	14.0	14.6	12.7	12.4
	θ/°	−169.6	171.9	−163.4	−176.3	−178.6	168.4	−168.4	178.5	176.3	163.4	−171.9	169.6

## Data Availability

Data are contained within the article.

## Appendix A

The thermal expansion coefficients and the temperature-induced modulus of Ti-6Al-4V and 2024-T351 are provided by Commercial Aircraft Corporation of China, as shown in Table A1. The specific material properties are listed in the below table. The elastic modulus of Ti-6Al-4V at −55 °C is 102% of that at ambient, and the elastic modulus of 2024-T351 at −55 °C is 104% of that at ambient. The thermal expansion coefficient of the two metal materials has little difference at −55 °C and ambient, respectively.

**Table A1:** The thermal expansion coefficients and the temperature induced modulus at ambient and −55 °C.

Materials	Temperature/°C	E/MPa	α/($10^{-6}\cdot {{}^{\circ}C}^{-1}$)
Ti-6Al-4V	21.11	116,521	8.82
	−55	118,851	8.46
2024-T351	21.11	73,777	22.17
	−55	76,728	21.78

Different seed numbers were selected, including 24, 28, 32, 36, and 40, to calculate using 40 multiple processors with ABAQUS. Table A2 shows that the maximum stress tends to be stable when the number of seeds exceeds 32, the bolt load on R4C1 changes little, and the calculation time increases obviously as the number of seeds increases. Considering computational accuracy and resources, we selected that the mesh size of the hole edge was at least 1.2 mm in the article.

**Table A2:** Calculation results on bolt R4C1 in different seed numbers at room temperature when mechanical load is 600 kN.

Seed Numbers	Maximum Stress at the Hole Edge/MPa	Bolt Load/kN	Calculation Time/s
24	195.4	19.2	5164
28	203.5	19.5	6230
32	210.6	19.7	7489
36	212.3	19.8	9024
40	213.5	19.8	10,326

The preload value to the bolts is listed in Table A3. The values in the table are the tightening torques applied to bolts of different diameters during machining of the specimen.

**Table A3:** Tightening torques and preload values to bolts of different diameters.

Diameter/mm	Torque/Nm	Preload Value/kN
11.1125	63.55	19.1
12.7	87.56	23.0
14.2875	121.46	28.3

During the test, both ends of hybrid panel are hinged with the fixture through three studs of 60 mm diameter, and the nut is tightened through the thread of the stud end as shown in Figure A1. The distance is 190 mm, and the fixture does not interfere with the bolt of the test piece. So, both ends of the hybrid panel are constrained along the x-direction, which does not affect its movement under the action of the mechanical load.

## Appendix B

The simulation model is established in the commercial finite software ABAQUS 2020. The model’s details, including boundary conditions, contact property, metal elastoplasticity and progressive damage failure of composite materials, are realized by the built-in functions of the software. The Hashin material failure criterion [28,29,30] considers the influence of four failure modes: fiber tensile failure, fiber compression failure, matrix tensile failure and matrix compression failure.

fiber tensile failure ${\hat{\sigma }}_{11}\;\geq \;0$:
  (A1) $${\left(\frac{{\hat{\sigma }}_{11}}{X^{T}}\right)}^{2}+\alpha {\left(\frac{{\hat{\sigma }}_{12}}{S^{L}}\right)}^{2}=1,$$
fiber compression failure ${\hat{\sigma }}_{11}\;<\;0$:
  (A2) $${\left(\frac{{\hat{\sigma }}_{11}}{X^{C}}\right)}^{2}=1,$$
matrix tensile failure ${\hat{\sigma }}_{22}\;\geq \;0$:
  (A3) $${\left(\frac{{\hat{\sigma }}_{22}}{Y^{T}}\right)}^{2}+{\left(\frac{{\hat{\sigma }}_{12}}{S^{L}}\right)}^{2}=1,$$
matrix tensile failure ${\hat{\sigma }}_{22}\;<\;0$:

(A4) $${\left(\frac{{\hat{\sigma }}_{22}}{2S^{T}}\right)}^{2}+\left[{\left(\frac{Y^{C}}{2S^{T}}\right)}^{2}-1\right]\frac{{\hat{\sigma }}_{22}}{Y^{C}}+{\left(\frac{{\hat{\sigma }}_{12}}{S^{L}}\right)}^{2}=1,$$

where $X^{T}$ is longitudinal tensile strength, $X^{C}$ is longitudinal compressive strength, $Y^{T}$ is transverse tensile strength, $Y^{C}$ is transverse compressive strength, $S^{T}$ is transverse shear strength, $S^{L}$ is longitudinal shear strength, and α is the influence coefficient of shear stress on the initial tensile criterion of fiber.
